# Group motivational intervention in overweight/obese patients in primary prevention of cardiovascular disease in the primary healthcare area

**DOI:** 10.1186/1471-2296-11-23

**Published:** 2010-03-18

**Authors:** Juan José Rodríguez Cristóbal, Josefa Ma Panisello Royo, Carlos Alonso-Villaverde Grote, José Ma Pérez Santos, Anna Muñoz Lloret, Francisca Rodríguez Cortés, Pere Travé Mercadé, Francisca Benavides Márquez, Pilar Martí de la Morena, Ma José González Burgillos, Marta Delclós Baulies, Domingo Bleda Fernández, Elida Quillama Torres, representing the IMOAP group

**Affiliations:** 1 Medicina, Area Básica Salud Florida Sud, Parc dels Ocellets, L'Hospitalet del Llobregat, Barcelona, Spain; 2 Departamento de Medicina, Hospital de Igualada, Barcelona, Spain; 3 Institut Català de Ciències Cardiovasculars, Centre superior d'Investigacions Científiques, Barcelona, Spain; 4 Departament mèdic, Solvay Pharma, Barcelona, Spain; 5 Unitat de Salut Mental Collblanc, L'Hospitalet del Llobregat, Barcelona, Spain; 6 Laboratorio Clínico L'Hospitalet. Institut Català de la Salut, Centre Assistència Primària Just Oliveras, L'Hospitalet de Llobregat, Barcelona, Spain; 7 Area bàsica de la Salut Rio de Janeiro, Barcelona, Spain; 8 Hospital de Viladecans, Viladecans, Barcelona, Spain

## Abstract

**Background:**

The global mortality caused by cardiovascular disease increases with weight. The Framingham study showed that obesity is a cardiovascular risk factor independent of other risks such as type 2 diabetes mellitus, dyslipidemia and smoking. Moreover, the main problem in the management of weight-loss is its maintenance, if it is achieved. We have designed a study to determine whether a group motivational intervention, together with current clinical practice, is more efficient than the latter alone in the treatment of overweight and obesity, for initial weight loss and essentially to achieve maintenance of the weight achieved; and, secondly, to know if this intervention is more effective for reducing cardiovascular risk factors associated with overweight and obesity.

**Methods:**

This 26-month follow up multi-centre trial, will include 1200 overweight/obese patients. Random assignment of the intervention by Basic Health Areas (BHA): two geographically separate groups have been created, one of which receives group motivational intervention (group intervention), delivered by a nurse trained by an expert phsychologist, in 32 group sessions, 1 to 12 fortnightly, and 13 to 32, monthly, on top of their standard program of diet, exercise, and the other (control group), receiving the usual follow up, with regular visits every 3 months.

**Discussion:**

By addressing currently unanswered questions regarding the maintenance in weight loss in obesity/overweight, upon the expected completion of participant follow-up in 2012, the IMOAP trial should document, for the first time, the benefits of a motivational intervention as a treatment tool of weight loss in a primary care setting.

**Trial Registration:**

ClinicalTrials.gov Identifier: NCT01006213

## Background

### Introduction

Overweight and obesity are growing health problems throughout the industrialised world. If not controlled, it then will continue to contribute to the burden of non-transmitable diseases, which is increasing constantly. There is currently clear scientific evidence of the association of obesity and a large number of diseases and their manifestations, including: diabetes, hypertension, dyslipidemia, cardiovascular events and inflammatory markers such as plasma fibrinogen levels and C-reactive protein [1,2].

Overall mortality for cardiovascular causes increases with weight [3,4] and, in the Framingham study, obesity was seen to be an independent risk factor for CVR regardless of other risk factors such as type 2 diabetes mellitus, dyslipidemia and smoking [5,6]; at the same time, overweight and obesity in adults significantly reduces life expectancy, in both women and men and smokers and non-smokers [7].

The prevalence of obesity is increasing continuously in developed countries, causing a serious public health problem. According to the SEEDO'2000 study, the figures for the prevalence of obesity in Spain (BMI>30) are 14.5% (13.3% men and 15.7% women) while the overall overweight and obesity figure (BMI>25) is 53.5% [8,9].

When treating overweight and obesity, it is considered essential that diet should be included in a general programme, together with physical exercise, lifestyle changes and psychological support. However, although significant weight loss can be relatively easily achieved in the short term, its maintenance is very difficult to get, is for this that the research that could help these patients is so important. Drug treatment of obesity should not be used as an isolated therapy, but together with other basic therapies, such as diet, physical activity and psychological support. It should be prescribed to patients with BMI>30 or >27 if associated with co-morbidities, such as diabetes mellitus, arterial hypertension, dyslipidemia, sleep apnoea syndrome [10-13].

Weight loss is a healthcare need that leads to significant savings in the estimated cost of obesity and the diseases associated to it, both directly and indirectly, which amount to more than 2,500 million euros per annum, a figure that accounts for almost 7% of total healthcare costs [14,15].

A variety of individual and group psychological therapies have been used in weight loss. In a study conducted in 20 primary healthcare centres in Great Britain, researchers evaluated the efficacy of a motivational interview to modify fat intake, physical activity and smoking in 883 patients at high risk of cardiovascular disease, obtaining a benefit in the intervention group [16].

Motivational intervention alone, in obese patients, has been shown to be effective, even in patients who only took a drug (sibutramine) for weight reduction [17]. But psychological interventions are particularly useful when combined with dietary and exercise strategies. In this review of 36 clinical studies, 26 were conducted in the United States, and only one in Spain, in diabetic obese patients [18,19].

Other studies using motivational interviews have been shown to be effective for giving up smoking [14,17], reducing alcohol intake [20,21] and improved adherence to treatment strategies in diseases as common as arterial hypertension, diabetes mellitus and bronchial asthma [22].

When making lifestyle changes in the adult population, the interventional methods are complex, because individuals tend to cling to what is familiar, even though this may be putting their health at risk [23].

All changes involve some elements that evolve and others that remain stable. The aim is to help patients make them part of their lives without affecting what they feel is essential. However, it is important to help them perceive these as necessary and beneficial so that a series of changes they make will give them an awareness that their health depends on the type of habits they change, and that these can be taken as an exercise that will improve their health and well-being[21].

However, there are no studies in our primary care environment, in cardiovascular disease, that assess the efficacy of an intervention based on a group motivation interview about weight reduction in overweight and obese patients, and the maintenance of the weight achieved over time.

Studies are required among our patients to confirm the efficacy observed in other populations and the feasibility of the group motivation intervention in overweight and obese patients, with a view to suggesting its application in the primary care setting as a tool for dealing with this health problem.

For these reasons, a study has been proposed, to compare the efficacy of the healthcare professionals' usual practices, together with a motivational group intervention vs an isolated traditional intervention on weight loss and its maintenance in overweight and obese patients (IMOAP). The purpose of this report is to present the design of IMOAP trial.

## Methods

### Study overview

This study will be randomised, multi-centre interventional, in overweight and obese patients, with a follow-up of 26 months.

The overall goal of IMOAP study is to assess whether the efficacy of the healthcare professionals' usual practices, together with a motivational group intervention (delivered by a nurse trained by an expert psychologist), is more effective than an isolated traditional intervention on weight loss and its maintenance in overweight and obese patients. Furthermore, the study will evaluate whether this result has a positive impact on quality of life, changes in eating habits, and a reduction in the associated cardiovascular risk factors and overall cardiovascular risk.

Table 1 presents the timeline of the study. Protocol development, external review occurred over an initial 4-month period beginning in 2007.

**Table 1:** Timetable of the IMOAP trial

Phase	No. of Months	Calendar Dates	Trial Activities
1	4	01/07-04/07	Protocol development.
2	8	05/07-12/07	Acceptance to participate of Basic Health Areas.
3	2	01/08-02/08	Distribution and randomisation of the Basic Health Areas into both groups: Intervention/controls. For those BHA that will visit the intervention groups, training of nursing staff and coordinating the teams, creating templates and study protocols.
4	30	03/08-09/10	Main trial recruitment and follow-up
5	26	10/10-12-12	Follow-up only
6	4	12/12-04/13	Analysis and reporting

A second phase had the purpose of the acceptance to participate of Basic Health Areas.

For this, the coordinators have contacted the Basic Health Areas, to explain the protocol and confirm their participation. After this period, it has been the distribution and randomisation into the study groups: Intervention/controls. For those BHA that will visit the intervention groups, it has been done the training of nursing staff and coordinating the teams, creating templates and study protocols.

The training of the nursing staff has consisted in a basic training strategy, initially aimed at nurses and focusing on group motivation for life-style changes in overweight and obese patients, because lack of motivation is a common cause of therapeutic failure. The training has consisted of a number of workshops where role play techniques have taken place to help assimilate the concepts taught. This workshop has been delivered at the BHA of the intervention group and written material delivered regarding the content. Secondly, a written guide has been created to standardise the information the nurses provide during the overweight and obesity check-up visits. The guide includes information about the disease, healthy habits and diet.

Randomization into the study began in first trimester 2008, with a recruitment goal of 1,200 participants. The intervention and main trial recruitment will take place over 30 months with the patients in the intervention and control groups.

Finally, four months before the end of the project the final laboratory tests will be performed and the data collected for statistical analysis and reporting.

The first patient was entered on September 2008. The final visit for the last randomized participant is planned for the last trimester 2010, with final study reports expected in the first trimester of 2013.

### Selection of the study subjects

The IMOPAP inclusion and exclusion criteria are presented in Table 2. These criteria have been established to identify a trial population with overweight and obesity, and with sufficient statistical power with the proposed sample size. They will be included sequentially, from the beginning of the study. To avoid possible bias in the selection of patients, and not overburden the nursing staff, patient recruitment and follow up will be rolled out in stages during the first six months of the study to the first two patients who meet the study inclusion requirements and who present none of the exclusion criteria. This will carry out superior quality control, using smaller sample size than would be possible if we randomised the patients. The institutional review Ethics Committee of Jordi Gol i Gurina Foundation in Barcelona approved the conduct of the study. In addition, the study has been done in compliance with the Helsinki Declaration. Each IMOAP participant has provided written informed consent using procedures reviewed and approved by the EECC review board.

**Table 2:** Inclusion and exclusion criteria

*Inclusion criteria*
1. Overweight (BMI>25) and obese (BMI>30) patients of both genders, registered in the medical history (MH) or recently diagnosed.
2. Aged between 30 and 70 years
3. Agreement to participate in the study

*Exclusion criteria*
1. Patients with severe clinical pathology (bedridden, dementia, advanced neoplasia, etc.)
2. Patients with secondary obesity (hypothyroidism, Cushing's disease, etc)
3. Patients with severe sensorial disorders capable of interfering with the motivational intervention (severe, uncorrected deafness, severe visual deficit, etc).
4. Patients with serious psychiatric disorders

### Measurements

A wide range of interview, physical examination, and laboratory data are being collected (Table 3), with the frequency of measurement varying by group assignment, but at least at baseline, at one year and at the end of the trial. The weight measurement will be done always in the same conditions (the patient wearing underwear only, barefoot, and at the same time of day). Body Mass Index (BMI) will be calculated as: Weight in kilos divided by height in square metres W (kg)/H (m2). Waist measurement will be defined as the midline between the edge of the lowest rib and the iliac crest alongside the anterosuperior iliac spine, in cm. The cardiovascular risk factors will also be assessed: Hypertension will be defined as either blood pressure readings of above 140/90 on three occasions, (in diabetics, above 130/80); or patients receiving treatment for hypertension or with a clinical diagnosis of hypertension; Diabetes Mellitus: By case history or two prandial glycaemia readings > 126 mg/dl; -Smoking: packets year.

**Table 3:** Measures

1. Questionnaires
a. Sociodemographics: age, ethnicity, sex.
b. Detailed initial medical history; abbreviated interval history focused on eligibility criteria, CVD, smoking status and diabetes mellitus. Inter-current processes: diseases, level of care they have received, start and end of the process.
c. Concomitant medications:
d. Quality of life and food assessment through SF questionnaire 36 validated in Spain by Alonso [24]
e. Dietary survey, 3 day record, (quantitative and qualitative) at the beginning and end of the study [25].
f. Questionnaire drafted specifically for this study, described in table 4.
2. Physical examination measures
a. Anthropometric measurements: standing height in centimetres (cm), weight in kg, waist circumference
b. BP and pulse
3. Laboratory measures
a. Fasting serum glucose
b. Fasting lipid panel (Total Cholesterol, HDL-Cholesterol, LDL- Cholesterol)

Quality of life and food assessment through SF questionnaire 36 validated in Spain by Alonso [24], and dietary survey, 3 day record, (quantitative and qualitative) at the beginning and end of the study [25].

A questionnaire, drafted specifically for this study, and administered by the participating nurse, gathers the variables mentioned.

### Outcomes

The primary endpoint for the IMOAP is to assess whether the efficacy of the healthcare professionals' usual practices, together with a motivational group intervention (delivered by a nurse trained by an expert psychologist), is more effective than an isolated traditional intervention on weight loss and its maintenance in overweight and obese patients.

This will be calculated as the percentage of patients reducing their weight by 5% and maintenance over time. The strategies for facing change and working in the groups [26-28] consist in providing information and practical advice on modifying eating habits, starting or increasing regular physical activity, lifestyle changes, functioning of the metabolism, management of the obesity and its complications, smoking as a cardiovascular risk factor, food diary, shopping methods and, finally, the number of calories to ingest.

### Formation of the patient groups

Groups will be made up according to the following criteria: mixed, women and men, aged between 30 and 70 years, and a maximum 15 and minimum 7 members per group. Each nurse could manage 3 different groups (1 per month). The random assignment of the intervention will be done at the primary care Basic Health Areas (BHA), in L'Hospitalet de Llobregat, neighbouring areas and Barcelona city. Two groups will be formed in separate centres, one of which will receive the motivational group intervention (intervention group) and the other the standard treatment (control group). To prevent possible bias, the BHA located in the same building will be assigned to the same group (control or intervention). The participating healthcare professional (nurse) will select the first two patients with visits during the pre-established recruitment period, who met the study criteria.

### Intervention control group

Participants will follow the usual intervention, according to the protocols in each centre, as it is described elsewhere [8]. The visits will be done every 3 months, including advice on life-style changes, physical exercise, hypo caloric diet, containing 1,200-1,500 kcal and anthrometric measurements (weight, height and waist perimeter). Follow up of blood tests by a healthcare professional will be done at the beginning, at one year and at the end of the study. In additional file 1 it is described the content of their visits.

### Motivational intervention in the intervention group

Participants will follow the usual intervention, the same as in the control group, plus a group motivational intervention every 15 days, at the initial weeks 1 and 12 of the intervention, following the LEARN (Lifestyle, Exercise, Attitudes, relationships and Nutrition) programme [12] and then monthly at weeks 13 and 32, following the instructions of the Weight Maintenance Survival Guide programme [13]. Each session would last for one hour, for a 26 month follow up period, with a total of 32 interventions, as described in Table 4. Request for blood test at the start, at one year and at the end (3-year study duration). In additional file 2 it is described contents of the visit.

**Table 4:** 32 group sessions of the intervention.

In **sessions 1 to 4** (fortnightly) we should raise awareness among the patients of the benefits of changing their habits, which will allow us to move from the **"pre-contemplation" **to the **"contemplation" **stage.
To do this we must provide them with ample information about the benefits of change, the recommended diets which will be dealt with individually with the nurse or doctor, physical exercise, etc.
This will be reinforced with the instructions given to individuals.
1. We will explore their fears, earlier failures, worries and difficulties that prevent them from taking the decision.
2. We will help sort out their doubts about unhealthy behaviour or habits to encourage life style changes towards a healthier life.
3. We will give them the tools they need to face situations where their usual strategies have failed.
4. We will encourage the patient to move towards the desire to change, attempting to help them recognise and take care of their present and future problems and strengthening their perception of the effects.
5. We will work on the patients' resistance.

In **sessions 5 to 12** (fortnightly) we will have dispelled the patients' doubts and they will feel motivated to make a change to healthy habits and be committed to continuing with the programme.
We will then move into the "**determination" **stage
**1. **Reinforce the patient's self-motivational affirmations.
**2. **Point out the positive and negative aspects of the behaviour to suppress, the old and the new to be acquired.
**3. **Elaborate on these by asking for examples, clarifying the how, when, why...
**4. **Use extremes and image the best and worst results of the changes as well as their possible consequences.
**5. **Look backwards and/or forwards. Visualise with the patient what it was like before and how he/she thinks, he/she is going to feel once he/she has given up this unhealthy habit.
**6. **Explore the feelings associated with the change (fear, anxiety, etc)
**7. **Explore if he has help and support from a partner/family.
**8. **Strengthen the active role the patient has in the change.
**9. **Understand the resistance to change.
**10. **Help develop strategies.
**11. **Develop a joint action plan
**12. **Combat feelings of loss caused by the change.

In **sessions 13 to 32** (monthly) work with **"changes" **and **"maintenance"**.
**1. **Bolster the capacity to change
**2. **Encourage the patients to give their own reasons.
**3. **Increase the consciousness towards change.
**4. **Understand the stress these changes in habits cause in the patients
**5. **Reinforce the benefits of change.
**6. **Bolster self-efficacy.
**7. **Support the changes.
**8. **Boost each member's capacity to make a change.
**9. **Understand the patients' emotions and thoughts.
**10. **Give empathetic responses.
**11. **Assist with the expression of feelings and emotions.
**12. **Prevent relapses through exploration
**13. **Identify risky situations and find strategies for dealing with them.
**14. **Support the work and efforts made and the benefits of change and maintenance of their decision.

From 1 to 12, fortnightly sessions, from 13 to 32, monthly sessions.

### Objectives of the psychological support

Table 5 describes the main objectives proposed for the interventional group, aimed to facilitate the awareness and compliance with a series of requirements for lifestyle changes that will reinforce the medical follow up by creating intervention groups with psychological support to be conducted by nursing staff (previously trained by expert psychologists), [26,27]:

**Table 5:** Objectives of the physcological support:

•Give patients the opportunity to explain their difficulties, to achieve the required objectives.
•Share their experiences and feelings with one another from their group team.
•Enable the appearance and elaboration on the anxiety that arises with lifestyle changes.
•Reinforce the changes made.
•Explain the benefits of these new attitudes.
•Clear up any doubts.
•Induce shared positive reinforcement.

The Stages through which the patient passes before taking the decision to change his or her habits, following the Prochaska model [29], are described in Table 6.

**Table 6:** Stages in the intervention group:

A) Pre- contemplation	1. Give the patient plenty of advice 2. Guide the patient in relation to the programme 3. Get commitment through the motivational interview. 4. Evaluate the willingness and motivation to make changes.
B) Contemplation	1. Evaluate willingness and motivation to make the changes 2. Get a realistic commitment through the motivational interview 3. Reach an agreed decision to change behaviour and habits.

C) Preparation for action	1. Create an action plan within the group, adapting this to individual possibilities 2. Invite the family to collaborate.

D) Action	1. Introduce initiation of prevention of relapses during the group sessions. 2. Introduce advice, educational activities about healthy eating habits and smoking. 3. Include reinforcement for achievements.

E) Maintenance	1. Introduce maintenance of prevention of relapses during the group sessions 2. Maintain the reinforcement for the achievements made, highlighting the health benefits.

### Sample size calculation

The number of subjects necessary to divide into two independent groups has been calculated. With the standard intervention, a weight reduction of 5% of weight is expected (P1) [30,31]. It was predicted that the experimental intervention would lead to 75% of the patients reducing their weight by 5%. It will be assumed an alpha risk of 0.05 (bilateral hypothesis) and a beta risk of 0.20 (a potential 80%). If the interventions will be assigned individually, in other words, per patient, the number of patients in each group would be 328 (756 in total). If we assume a percentage loss of 20% and apply a correction according to the formula Na = N [1/1-R)] where N is the theoretical number of subjects, Na is the corrected number of subjects and R is the expected proportion of loss, a total of 946 patients would be necessary. Given that the assignment of the interventions will be done by BHA, we had to increase the sample size because of the design effect [28]; if 24 BHA (clusters) were to participate, 12 per type of information, it would be K = [m(1-CCI)]/[Z-(CCI × m)] where K = number of subjects per cluster, m = 473 patients per intervention, Z = number of clusters per type of intervention = 12 and ICC = intra-class correlation coefficient. We assumed that the basic ICC for the percentage of obese patients who would reduce their weight by 5% was 0.005. The number of patients in each BHA (Cluster) would be 50. 50 subjects × 12 BHA = 600 patients per arm or type of intervention. 600 subjects × 2 interventions = a total of 1,200 patients.

### Statistical analysis

A descriptive statistical analysis will be made according to the variables. For the quantitative variables, using the mean and standard deviation, for the qualitative ones, proportions will be used. The data analysis will include an evaluation of the initial comparability of the patients in the two types of intervention using bivariate techniques: The Chi-Square for the proportions and, in the case of the mean, the Student's t-distribution or its non-parametric equivalent when necessary, by point estimation with a confidence interval of 95%.

The hypothesis to compare will be the null hypothesis, defined as equal efficacy of the usual and experimental interventions (both interventions achieved a weight loss of 5% in the same proportion of patients). The possible difference in efficacy between the interventions will be carried out through an analysis based on the individual values of the patients using multilevel statistical techniques [32].

## Discussion

Overweight and obesity, serious and growing health problems, are not receiving the attention they deserve from primary care practitioners. An additional problem related to weight loss, if achieved, is its maintenance.

The study IMOAP analyses the management of overweight and obesity, in a group motivational intervention, managed by a nurse trained by an expert psychologist, in addition to usual practice, to see if this intervention is more efficient than the latter alone in the treatment of overweight and obesity in relation to percentage weight loss and maintenance. Also, if so, if it would it be possible to reduce the prevalence of classic and emerging cardiovascular risk factors that are related to overweight and obesity, and, in consequence, cardiovascular conditions.

The results of IMOAP trial should provide substantial direction regarding weight loss management at a primary care, and it could be introduced as a basic treatment tool for overweight and obese patients.

## Competing interests

AML acts as a scientific advisor for Solvay Pharma.

All other authors declare that they have no competing interest.

## Authors' contributions

JJRC formulated the research question, designed the study and supervised its conduct together with JPR, CAV and JPS. AML has been involved in drafting the manuscript. FRC, FBM, PMM, MJGB, MDB, DBF and EQT have monitored the nurses. All the authors approved the final manuscript.

## Pre-publication history

The pre-publication history for this paper can be accessed here:

http://www.biomedcentral.com/1471-2296/11/23/prepub

## Supplementary Material

Additional file 1 Control definite Click here for file

Additional file 2 Intervention Click here for file
