# Supplementary material for: Group motivational intervention in overweight/obese patients in primary prevention of cardiovascular disease in the primary healthcare area
Source: BMC Fam Pract. 2010 Mar 18;11:23. doi: 10.1186/1471-2296-11-23 (PMC2858126; doi:10.1186/1471-2296-11-23)
Supplement: Additional file 1 — Control definite [file 1471-2296-11-23-S1.DOC]

Additional file 1.

| **Content of Visit 1 of the Control Group (Visit 1c)** |
| --- |
| To confirm compliance with all the criteria and assess their inclusion, patients will be asked to come to the clinic for the specific purpose of informing them about the study, asking for their collaboration and, if they agrees, completing in the informed consent document for participation in the study. |
| Once this consent has been obtained (**ESSENTIAL**) the following information will be gathered (completing VISIT 1c record document): |
| 1. Information about the patient's cardiovascular status by taking a medical history of the presence of CV risk factors, associated clinical disease, dietary and health measures taken and the current medication prescribed. |
| 1. Physical examination -weight, height, body mass index calculation (weight in kilos divided by the squared height in metres- , measurement of the abdomen and blood pressure reading according to WHO guidelines |
| 1. Record the presence of criteria for inclusion and the Basic Health Area of the criteria for exclusion. |
| 1. The patient is given an appointment to come back in three months’ time to collect anthropometric information. |
| From this moment forth, the nurse will take the measures and do the controls as he/she sees fit, **according to her own criteria**. The patients medical history must be handled in the usual way at **each and every visit** made by the patient for control purposes, as well as any changes with regard to health and dietary advice and anthropomorphic parameters. |
| **Final assessment visit for the control group (Visit 9).** |
| This must take place at 32 weeks ± 2 weeks after visit 1 c The content of this visit must be identical to that in Visit 1c. The results of the laboratory tests ordered at the previous visit will be reviewed and analysed. |
