# Supplementary material for: Group motivational intervention in overweight/obese patients in primary prevention of cardiovascular disease in the primary healthcare area
Source: BMC Fam Pract. 2010 Mar 18;11:23. doi: 10.1186/1471-2296-11-23 (PMC2858126; doi:10.1186/1471-2296-11-23)
Supplement: Additional file 2 — Intervention [file 1471-2296-11-23-S2.DOC]

**Additional file 2.**

**Content of Visit 1 of the Intervention Group (1^st^ Visit)**

To confirm the compliance with all the criteria and assess their inclusion, patients will be asked to come to the clinic for the specific purpose of informing them about the study, asking for their collaboration and, if they agree, filling in the informed consent document to participate in the study.

Once this consent has been obtained (**ESSENTIAL**) the following information will be gathered (and filled in on the VISIT 1 record document):

1. Information about the patient's cardiovascular status by taking a medical history of the presence of CV risk factors, associated clinical disease, dietary and healthy measures taken and the current medication prescribed.
2. Physical examination -weight, height, body mass index calculation (weight in kilos divided by the squared height in metres-, measurement of the abdomen and blood pressure reading according to WHO guidelines.
3. Record the presence of criteria for inclusion and the Basic Health Area of the criteria for exclusion.

The time when the scheduled protocolised visit ends is recorded. The date when the patient will attend the 2nd scheduled visit is recorded.

**Content of the follow-up visits (Intervention Group Visits 2 to 32 inclusive)**

The content of the follow up visits will be essentially the same, with the exception of the final follow up visit, which will be visit 31, where the laboratory tests will be ordered.

If the patient does not attend: Record of NON-ATTENDANCE

- In this case, an attempt will be made to locate the patient by telephone during at least the following seven days subsequent to her/his failure to appear, making at least three attempts to locate him/her on different days, to ascertain the reason for his/her failure to appear. If there is a justifiable reason why he/she did not attend or he forgot and the patient wants to continue with the study, he/she will be given a new appointment soon afterwards.

If it is not possible to locate him or the patient expresses the desire not to continue: END OF STUDY

If the patient comes to the next appointment, he will continue to be included in the study. If the patient who did not appear is excluded from the study, he will subsequently treated/seen at the usual clinic.

**Final assessment visit for the intervention group. (Visit 32)**

The content of the final visit will be identical to the follow up visits, although the results of the biochemical tests ordered at visit 31 will be reviewed and recorded. Subsequent visits from this time on will be at left to the criteria of the researcher. The patient will be informed that the study has ended.
